# Dr. Alder, on Quarantines, Contagion, and Fevers

**Published:** 1808-02

**Authors:** Thomas Alder

**Affiliations:** Edinburgh


### To the Editors of the Medical and Phyſical Journal.

GENTLEMEN,

I Am much obliged to you for reminding me in your Journal for November, "that the non-contagion doctrines have been familiar in America some time," as it gives me both an opportunity to state my claim, of being the original author of them, and at the same time, gives strong confirmation of their truth: for, certainly, the American practitioners ought to be much better acquainted with the causes of fevers than the British, so far as a knowledge of causes can be obtained by witnessing their operation, fevers being infinitely more prevalent and mortal in America than they are here. And I believe it is true, that nineteen of twenty of the practitioners in America, now believe that fevers *do not* arise from contagion; at least, I have been so informed.—With respect to my claim to being the original author of this doctrine, I shall be more explicit afterwards; but I will trouble you, now with stating that I published a Treatise in April, 1796, to prove that bad air and other agents, (not including contagion,) cause not only the intermitting fever, but other fevers, by impeding the free passage of the blood through the lungs, and thereby obstructing its discharge from the venous into the arterial system; and in the same work, I said, I thought *no fevers* were caused by contagion. And I have ever maintained this doctrine from that time to the present; publishing many works, both to shew what *were* the causes of fevers, and what was *not* the cause of them.

In my last, I distinguished that kind of bad air which produces endemic fevers, from that which causes epidemics. I maintained that the latter is evolved chiefly by volcanic and other *terrestrial expirations*, and that from being light and abundant, it can be carried by the wind *to a great distance*, without losing its faculty of producing disease;—but that the former is formed in the place where the endemic prevails, and is in general so heavy, that it moves but little from the place where it is formed, and does not rise high up in the air: I then shewed why Britain was much more under the influence of *both* these kinds of bad air in former times, than it is at present; and consequently more afflicted, both with *epidemic* and *endemic* diseases.

1st. I shall consider *the sources of endemic diseases*:—in general, these are the gases evolved by putrefaction joined with moisture. It must appear at the first glance, that all these exist copiously in most places which are troubled with bad endemics; and that, *ceteris paribus*, they must be both more abundant in warm climates, and also take more effect on the constitution there; for heat is both favourable to putrefaction and to evaporation, and it hurries the circulation, so as to make it carry the blood up to the lungs very fast, and by that means occasions the necessity of a pure air; while, at the same time, it makes the constitution so irritable, that any thing wrong in it, is more severely felt.—Bad endemics, therefore, are most frequent in hot climates. In Egypt, there is a prodigious quantity of mud left every year by the Nile, in the plains and in the cisterns, which are formed in all the large cities on its banks; accordingly, we find every year, when this mud begins to putrefy, endemics appear. In Egypt too, and particularly near Alexandria, there are lakes, which in Autumn, generally are sufficiently dry to leave a great quantity of their mud exposed to the air; and at these times, putrefactious gases are emitted from it, and fevers arise in the neighbourhood. But the mud left by the Nile, generally, does not begin to putrefy till near the spring. Prosper Alpinus is very particular in enumerating the fevers and other diseases which clearly arise from these two sources principally; though the badness of the waters, on some occasions, may assist in producing them. These diseases are all either endemic or sporadic; they are, however, sometimes very numerous and fatal.

Observations like these may be made on our settlements on the banks of the Ganges, and on several places on the banks of the Euphrates. The Ganges has a prodigious quantity of mud at its sides, and in several places a great extent of muddy or marsh-land in its neighbourhood; and at the seasons when all this mud is left uncovered by any depth of water, and, therefore, exposed to the action of the sun and air, it sends out great quantities of noxious gases, and then remittent fevers begin, as fatal as any in Egypt, and often as much so as the plague itself is. Bussorah, on the Euphrates, is exposed to noxious effluvia from the putrefaction of mud, &c. which the water at certain times leaves uncovered; but here the putrefaction is said to be assisted, and the gases formed from it, rendered more abundant by salt being mixed with the mud: the fevers which arise from these gases are very numerous and mortal.

Our unhealthy settlements in Africa are rendered unhealthy healthy by putrefaction and moisture from a similar cause; and so indeed is the Dutch settlement at Batavia; our possessions in the West Indies, and the places in America which have been most subject to fever: the air in the Islands of the West Indies is found to be *very moist.*

2dly. *Causes of epidemic diseases.* As putrefaction and moisture are now pretty generally believed to be the chief causes of endemic fevers, and can in general be tolerably easily discovered when these diseases prevail, I shall now advert to the causes of *epidemics.*

When epidemics prevail, they generally are most fatal in places which are subject to *endemics;* therefore it is clear, the endemic air, (if I may be allowed the expression) greatly assists in producing the epidemic disease:—thus the *campsin* joins with the endemic noxious air of Egypt; the *harmattan* unites with that of our unhealthy settlements in Africa; and the *syroc* adds itself to any locally generated bad air, which may be in Italy. These are all noxious airs, which travel to a great distance; and there is no doubt I may call them *epidemic airs.* The *campsin,* or South hot suffocating wind, is so bad, that it has been called "poisonous:"—when it comes upon people a little South of Egypt, (that is, near its source) it is so powerful, that if they do not fall down, it *suffocates* them in a few minutes; and when they do, it nearly suffocates them; as Mr. Bruce and his companions, and many others, have found; and when it blows upon Egypt *for three days together,* it causes many deaths. Now this suffocating wind blows in those months which are most subject to the plague, viz. in the spring months; and it ceases to blow precisely at the time when the plague ceases. The endemic bad air of Egypt, and the epidemic, cease both at the same time, and both exactly at that time when the plague and all other acute diseases cease: for the Nile overflows about Midsummer, and covers all the mud that was putrefying; the carbonic acid watery vapour at the same time is precipitated; and the south hot suffocating wind gives place to the Etenæ, or * cooling pure winds of the north; and then the plague and all other bad fevers cease; these all happen within a few days of each other, and all about the time of Midsummer. Several historians agree in this account, and I do not find any one that contradicts it; but most of them give their relations in such a

 way,

* This is very opposite to Dr. Blane's conjectures, way, that we can scarcely avoid being misled by them. Finding the dew which always falls then favourable to vegetation, and finding too the plague and all other acute diseases cease as soon as this dew has fallen, they most of them join with the natives in saying, *this dew stops the plague!* But it appears quite evident to me, that while it was suspended in the air in vapour, it was one chief cause of the air being unhealthy. It seems to be precipitated by the air growing much cooler from the change from a hot south to a cool northerly wind. That it is an acid, is proved by its having rusted Mr. Bruce's quadrant in the short space of one night; and that it is the carbonic acid, (joined I mean with water) seems demonstrated by its being favourable to vegetation. It is true, some French chemists have made experiments with the air of Egypt, and did not detect much carbonic acid in it; but I do not maintain that much carbonic acid exists in the air of Egypt, *in all places, and at all times;* and if I were to allow these eudiometric experiments proved the air *they measured* did not contain much carbonic acid, still it must be recollected, these chemists were philosophers enough to keep out of the way of the plague, and of every mortal (which they would consider "*contagious*") fever; and that it requires different eudiometric experiments from any which have yet been made, to ascertain the degree of purity of the air.—I intend, however, to allow, that several of these experiments, if accurate, have been sufficient to estimate how much carbonic acid there has been in it.

It appears, the *harmattan* is almost equally as morbiferous an air as the *campsin;* and from the direction of both, and several other circumstances, there can be little doubt they both come from the same source, viz. Æthiopia. And it is very remarkable, that not only the plague now comes in general into Egypt from the same quarter, but that almost all those terrible plagues which are mentioned by ancient authors, came from the same place; that is, they first attacked Æthiopia, then upper Egypt, then lower Egypt, or the contiguous countries, and so went forwards progressively; but sometimes made a circuit.

The *syroc* of Italy is a noxious wind; it makes people relaxed and ill, and very low-spirited; it blows in the spring months, and comes, according to my judgment, from the same source; but from travelling such a great distance, and passing over the Mediterranean, it has become much more diffused, and consequently much less injurious. I am informed this same kind of air reaches

Gibraltar;

Gibraltar; and from this I conclude, it frequently does injury in France, Portugal, and Spain, if not *in some degree* in England; but when it reaches us, it must be very diffused.

It thus seems to me, *these* are noxious winds, whose course and effects we can clearly trace, and that they probably arise from a volcano somewhere in Æthiopia. But the valuable history which I have so often quoted, (I mean Mr. Webster's) gives much clearer proof of the morbific operation of volcanic and similar gases, as it shews internal fires have been extremely active, at or about the period of almost every epidemic disease which has afflicted mankind for more than these last two thousand years. He proves the earth has often emitted gases, and sometimes flames, where there has been no volcano. It is true, Mr. Webster does not prove the eruption has always preceded the epidemic, or always been near it; but it must be recollected, when internal fires are very active, they often burst forth at several, and these very distant parts of the globe; and that gases very often are evolved long before flames, as well as often long after them; and indeed, that they sometimes have been evolved where no flames have succeeded. I would recommend it to every gentleman who wishes to form an accurate judgment concerning what these powers are, which have acted most fatally on the human constitution, to read this work.

I am, &c.

THOMAS ALDER.

*Edinburgh, Dec.* 7, 1807.

N. B. In my next, I shall endeavour to shew how those causes produce fever.

( To be continued. )

